# Association of the Triglyceride-Glucose (TyG) Index With Biochemical Parameters in Acute Ischemic Stroke: A Cross-Sectional Study

**DOI:** 10.7759/cureus.115247

**Published:** 2026-08-26

**Authors:** Bhavya Khattri, Jigar Haria, Vinod Singh

**Affiliations:** 1 General Medicine, Teerthanker Mahaveer Medical College and Research Centre, Moradabad, IND

**Keywords:** cholesterol, glucose, glycated hemoglobin, ischemic stroke, triglycerides

## Abstract

Background

Stroke carries substantial mortality and disability, making early prognostic risk stratification important. The Preadmission Comorbidities, Level of Consciousness, Age, and Neurological Deficit (PLAN) score provides a validated bedside approach to estimating adverse prognostic risk in acute ischemic stroke (AIS). The triglyceride-glucose (TyG) index is a simple, inexpensive marker derived from routinely available fasting triglyceride and glucose measurements and may reflect underlying metabolic dysfunction. However, its relationship with prognostic risk assessed by PLAN, and whether this association differs according to diabetes status, remains insufficiently characterized.

Objectives

This study aims (1) to assess the bivariate correlation between the TyG index and selected biochemical parameters in patients with AIS and (2) to examine the association between the TyG index and the PLAN prognostic score according to diabetes status.

Materials and methods

This cross-sectional study included 71 consecutive adults with AIS admitted to a tertiary care hospital in India between October 2024 and March 2026. Demographic, clinical, and laboratory data were collected using a structured pro forma. The TyG index was calculated as ln (fasting triglycerides (TG) (mg/dL) × fasting glucose (mg/dL)/2). The PLAN score was assessed during the initial clinical evaluation. Pearson or Spearman correlation analysis was performed based on the data distribution.

Results

The mean age of participants was 60.96 ± 12.20 years, with 39 (54.9%) males. Hypertension was the most frequent comorbidity (78.9%), followed by smoking (43.7%) and diabetes mellitus (35.2%). The mean TyG index and PLAN score were 9.36 ± 0.60 and 12.99 ± 4.19, respectively. The TyG index demonstrated significant positive correlations with fasting TG (ρ = 0.720), fasting glucose (ρ = 0.881), total cholesterol (ρ = 0.557), LDL-C (r = 0.504), and HbA1c (ρ = 0.348). In contrast, significant negative correlations were observed with hemoglobin (r = −0.242), HDL-C (ρ = −0.458), and serum albumin (ρ = −0.256) (all p < 0.05). A significant positive correlation was observed between the TyG index and PLAN score (r = 0.529, p < 0.0001), which remained significant after adjustment for clinical confounders (partial r = 0.540, 95% CI: 0.342-0.693; p < 0.0001). The association was also significant among diabetic (r = 0.608, p = 0.001) and non-diabetic patients (r = 0.520, p = 0.0002), with no significant TyG index × diabetes interaction (p = 0.306).

Conclusions

The TyG index showed significant associations with several metabolic and biochemical parameters and demonstrated a moderate positive association with PLAN prognostic risk among patients with AIS. This association persisted after adjustment for major clinical characteristics and was observed in both diabetic and non-diabetic patients, without evidence of effect modification by diabetes status. These findings suggest that the readily available TyG index may provide useful information regarding metabolic risk and its relationship with prognostic risk in AIS. However, the cross-sectional design precludes establishing causality or determining whether the TyG index independently predicts subsequent clinical outcomes. Larger prospective multicenter studies incorporating longitudinal outcomes and comprehensive adjustment for confounding are warranted.

## Introduction

Stroke is a major global public health concern and remains one of the leading causes of death and long-term disability worldwide [1]. Globally, there are an estimated 93.8 million prevalent cases of stroke and 11.9 million new cases each year, with stroke ranking as the second leading cause of mortality worldwide. Despite advances in acute stroke management, access to definitive reperfusion therapies remains limited, with hospital-based data suggesting that fewer than 5% of eligible patients receive mechanical thrombectomy [2]. In India, the burden is similarly substantial, with approximately 9,338,000 prevalent and 1,251,000 incident stroke cases reported in 2021, according to the Global Burden of Disease data [3].

The considerable mortality, disability, and socioeconomic burden associated with stroke highlight the importance of early identification of patients at greater risk of adverse prognostic outcomes [3]. In this context, the Preadmission Comorbidities, Level of Consciousness, Age, and Neurological Deficit (PLAN) score is a validated bedside prognostic tool developed to estimate the risk of adverse outcomes following acute ischemic stroke (AIS). It incorporates preadmission comorbidities, level of consciousness, age, and neurological deficits and has been used to predict outcomes including mortality and severe disability [4]. Importantly, the PLAN score is a prognostic prediction tool rather than a direct measure of stroke severity [4].

It has been hypothesized that identification of readily available biochemical parameters that correlate with prognostic risk may provide an additional, simple, and clinically accessible means of identifying patients at greater risk of poor outcomes. In this regard, the triglyceride-glucose (TyG) index, calculated from fasting triglyceride and glucose concentrations, has gained considerable interest because of its simplicity and ease of calculation [5]. It has shown a statistical association with AIS severity; however, its association with prognosis has been less extensively studied [5,6].

Additionally, diabetes mellitus is an important metabolic comorbidity in patients with stroke and is an established risk factor for both the development and adverse outcomes of stroke. Diabetic patients have an approximately 1.5-2-fold higher risk of stroke than non-diabetic patients, with risk increasing with longer disease duration [7]. Furthermore, hyperglycemia and associated metabolic disturbances may adversely influence outcomes following AIS. Given the close relationship between diabetes mellitus, insulin resistance, dysglycemia, and cerebrovascular outcomes, evaluating the TyG index separately among diabetic and non-diabetic patients may provide additional insight into its clinical relevance [8].

However, evidence on the relationship between the TyG index, biochemical parameters, and prognostic risk, as assessed by the PLAN score, in patients with AIS remains limited [9-17]. Furthermore, whether this association varies with the presence of diabetes mellitus has not been adequately explored [18]. Evaluating these associations may help determine whether an inexpensive, readily obtainable metabolic index, such as the TyG index, could provide additional prognostic information in AIS.

Thus, the present study was conducted with the primary objective of evaluating the association of the TyG index with biochemical parameters and prognostic risk, as assessed using the PLAN score, among patients with AIS. The secondary objective was to evaluate the TyG index and its associations with biochemical parameters and the PLAN score, stratified by the presence or absence of diabetes mellitus among patients with AIS.

## Materials and methods

This cross-sectional study enrolled 71 adult patients with AIS admitted to the Department of Medicine at a tertiary care hospital in India between October 2024 and March 2026. The study protocol was approved by the Institutional Ethics Committee of Teerthanker Mahaveer University (approval number: TMU/IEC/2024-25/PG/95), and all procedures were conducted in accordance with ethical standards. Eligible participants were informed about the study objectives and procedures, and written informed consent was obtained prior to enrollment.

Inclusion criteria were patients diagnosed with AIS based on clinical features of focal neurological deficit consistent with a vascular territory, as defined by the International Classification of Diseases, 11th Revision (ICD-11). Exclusion criteria were pregnant women; patients with renal insufficiency, liver cirrhosis, thyroid disorders, or malignancy; and patients with a BMI ≥45 kg/m².

The sample size was calculated based on the study by Garg et al. [6], which reported a mean TyG index of 9.26 ± 0.20 among patients with AIS. The required sample size was calculated using the formula $n = \frac{Z_{\alpha/2}^{2}\mathrm{SD}^{2}}{d^{2}}$​​​​​​, where n stands for the required sample size, $Z_{\alpha/2}$​ represents the standard normal deviate corresponding to a two-tailed significance level of 5% (1.96), SD represents the standard deviation (SD) of the outcome variable, and d represents the allowable margin of error. Substituting the relevant values into the formula yielded an estimated sample size of 62 participants. To enhance the precision and statistical robustness of the analysis, the study ultimately included 71 participants.

Baseline demographic and clinical data, including age, sex, smoking status, and comorbidities, were recorded using a structured data-collection form. The PLAN prognostic score, which incorporates preadmission comorbidities, level of consciousness, age, and neurological deficits, was assessed [19]. The PLAN score is a validated bedside prognostic prediction rule developed for patients with AIS. It incorporates preadmission comorbidities, level of consciousness, age, and neurological deficits to estimate the risk of adverse outcomes, including 30-day and one-year mortality and death or severe disability at hospital discharge [19]. The score was assessed during the patient's initial clinical evaluation following admission.

For laboratory investigations, 5 mL of venous blood was collected in a plain vacutainer and an additional 5 mL in an EDTA vial. Venous blood samples were collected during the initial evaluation of patients following admission for AIS. Fasting blood samples were used to estimate fasting blood glucose and the fasting lipid profile, which were subsequently used to calculate the TyG index.

The evaluated biochemical parameters included complete blood counts, fasting blood glucose, glycated hemoglobin (HbA1c), liver and kidney function tests, serum sodium and potassium levels, fasting lipid profile, and viral markers (HBsAg, anti-HCV, and HIV). The TyG index was calculated using the formula [6]:

$\mathrm{TyG\ index} = \ln\left[\frac{\text{fasting triglycerides (mg/dL)} \times \text{fasting blood glucose (mg/dL)}}{2}\right]$

The primary outcome was to determine the bivariate correlation of the TyG index with selected biochemical parameters and the PLAN score among patients with AIS. The secondary outcome was the correlation between the TyG index and the PLAN score among diabetic and non-diabetic patients.

Statistical analysis

All statistical analyses were performed using SPSS Statistics version 25.0 (IBM Corp. Released 2017. IBM SPSS Statistics for Windows, Version 25.0. Armonk, NY). Continuous variables were summarized as mean ± SD or median (interquartile range (IQR)), as appropriate, while categorical variables were presented as frequencies and percentages. The distribution of continuous variables was assessed before selecting the appropriate correlation test. Pearson's correlation coefficient was used for normally distributed variables, whereas Spearman's rank correlation coefficient was used when the assumptions for Pearson correlation were not met. The association between the TyG index and PLAN score was similarly assessed using bivariate correlation analysis, and the correlation coefficient (r) and corresponding p-value were reported. Regarding potential confounders, demographic and clinical variables, including age, sex, smoking status, and relevant comorbidities, were recorded as potential clinical factors but were not included in a multivariable adjustment model for biochemical parameters, as the primary statistical analysis consisted of unadjusted bivariate correlation analyses. However, the correlation between the TyG index and PLAN score was adjusted for these clinical confounders.

In terms of subgroups, diabetic and non-diabetic patients were compared with the independent-samples t-test (with Welch correction when unequal variances were identified using the F-test) for normally distributed variables, the Mann-Whitney U test for non-normal variables, and the chi-square test (or Fisher's exact test when an expected cell count was below 5) for categorical variables. The correlation between the TyG index and the PLAN score was then examined separately within diabetic and non-diabetic patients, and the difference between the two slopes was tested by a TyG index × diabetes interaction term in linear regression. A two-sided p-value <0.05 was considered statistically significant.

## Results

Baseline characteristics of study cohort

The age of the study participants ranged from 33 to 87 years, with a mean age of 60.96 ± 12.20 years. The study population comprised 39 men (54.93%) and 32 women (45.07%). Among the baseline characteristics, hypertension was the most common comorbidity, affecting 56 (78.87%) patients, followed by smoking history in 31 (43.66%), diabetes mellitus in 25 (35.21%), and heart failure in 15 (21.13%).

Analysis of viral markers revealed that 67 (94.37%) patients were non-reactive, while anti-HCV positivity was detected in 4 (5.63%) patients. None of the participants tested positive for cardiac biomarkers, as all 71 (100%) patients had negative CK-MB and troponin-I results.

The mean TyG index of the study population was 9.36 ± 0.60, with a median (IQR) of 9.20 (8.94-9.82). The mean PLAN score was 12.99 ± 4.19, while the median (IQR) was 13.0 (9.75-15.25).

When diabetic patients (n = 25) were compared with non-diabetic patients (n = 46), baseline characteristics were comparable between the groups, including age, sex, hypertension, smoking, and heart failure (all p > 0.05). The median (IQR) fasting glucose was significantly higher in diabetic patients, 186.0 (120.0-209.0) mg/dL vs. 112.0 (102.0-120.8) mg/dL in non-diabetic patients (p < 0.0001), while mean hemoglobin was significantly lower, 10.55 ± 2.16 g/dL vs. 11.58 ± 2.00 g/dL (p = 0.048). The mean TyG index was comparable between diabetic and non-diabetic patients (9.56 ± 0.75 vs. 9.26 ± 0.48; p = 0.077), as was the mean PLAN score (13.16 ± 3.94 vs. 12.89 ± 4.36; p = 0.798), as shown in Table 1.

**Table 1:** Comparison of baseline characteristics, biochemical parameters, TyG index, and PLAN score between diabetic and non-diabetic stroke patients Mean difference (diabetic minus non-diabetic): TyG index 0.30 (-0.03 to 0.64); PLAN score 0.27 (-1.82 to 2.36). Independent t-test for mean ± SD rows (Welch correction for the TyG index), Mann-Whitney U test for median (IQR) rows, chi-square test for proportions. SD: standard deviation, IQR: interquartile range, HbA1c: glycated hemoglobin, SGOT: serum glutamic-oxaloacetic transaminase, AST: aspartate aminotransferase, SGPT: serum glutamic-pyruvic transaminase, ALT: alanine aminotransferase, eGFR: estimated glomerular filtration rate, HDL-C: high-density lipoprotein cholesterol, LDL-C: low-density lipoprotein cholesterol, NT-proBNP: N-terminal pro-B-type natriuretic peptide, TyG: triglyceride-glucose, TG: triglycerides, TC: total cholesterol

Variable	Non-diabetic (n = 46)	Diabetic (n = 25)	P-value
Age (years), mean ± SD	60.98 ± 13.56	60.92 ± 9.56	0.985
Male sex, n (%)	26 (56.5%)	13 (52.0%)	0.715
Hypertension, n (%)	36 (78.3%)	20 (80.0%)	0.864
Smoking, n (%)	20 (43.5%)	11 (44.0%)	0.966
Heart failure, n (%)	10 (21.7%)	5 (20.0%)	0.864
Fasting TG (mg/dL), median (IQR)	170.0 (129.8-234.2)	172.0 (120.0-235.0)	0.814
Fasting glucose (mg/dL), median (IQR)	112.0 (102.0-120.8)	186.0 (120.0-209.0)	<0.0001
HbA1c (%), median (IQR)	5.8 (5.4-6.1)	6.0 (5.6-7.2)	0.159
Hemoglobin (g/dL), mean ± SD	11.58 ± 2.00	10.55 ± 2.16	0.048
Total leukocyte count (cells/µL), mean ± SD	9080.43 ± 3403.63	10012.00 ± 2504.22	0.234
Platelet count (×10³/µL), median (IQR)	179.0 (128.5-238.0)	211.0 (139.0-302.0)	0.248
Total bilirubin (mg/dL), median (IQR)	0.7 (0.5-0.9)	0.7 (0.4-0.8)	0.976
Direct bilirubin (mg/dL), median (IQR)	0.1 (0.1-0.2)	0.1 (0.1-0.2)	0.726
SGOT/AST (U/L), median (IQR)	39.0 (25.2-50.8)	39.6 (26.0-67.0)	0.496
SGPT/ALT (U/L), median (IQR)	28.0 (18.0-46.2)	36.0 (22.0-68.0)	0.101
Serum albumin (g/dL), median (IQR)	3.4 (3.0-3.8)	3.2 (2.8-3.8)	0.362
Serum urea (mg/dL), median (IQR)	40.5 (26.3-67.0)	37.0 (24.0-63.0)	0.643
Serum creatinine (mg/dL), median (IQR)	0.9 (0.6-1.3)	1.0 (0.7-1.5)	0.156
eGFR (mL/min/1.73 m²), median (IQR)	82.5 (55.2-99.8)	66.0 (47.0-95.0)	0.179
Serum sodium (mEq/L), mean ± SD	136.50 ± 6.84	137.20 ± 4.53	0.607
Serum potassium (mEq/L), mean ± SD	4.19 ± 0.67	4.28 ± 0.69	0.581
TC (mg/dL), median (IQR)	166.5 (131.2-215.0)	169.0 (150.0-268.0)	0.154
HDL-C (mg/dL), median (IQR)	40.0 (26.2-55.8)	46.0 (30.0-66.0)	0.134
LDL-C (mg/dL), mean ± SD	112.83 ± 38.86	126.40 ± 46.70	0.195
NT-proBNP (pg/mL), median (IQR)	334.5 (190.5-968.0)	679.0 (230.0-940.0)	0.324
TyG index, mean ± SD	9.26 ± 0.48	9.56 ± 0.75	0.077
PLAN score, mean ± SD	12.89 ± 4.36	13.16 ± 3.94	0.798

Correlation of the TyG index with biochemical parameters

Correlation analysis demonstrated significant positive associations between the TyG index and fasting TG (170 (127-237) mg/dL; ρ = 0.720; p < 0.0001), fasting blood glucose (118 (104.5-171.5) mg/dL; ρ = 0.881; p < 0.0001), HbA1c (5.9 (5.4-6.1)%; ρ = 0.348; p = 0.003), total cholesterol (TC) (167 (135-221.5) mg/dL; ρ = 0.557; p < 0.0001), and low-density lipoprotein cholesterol (LDL-C) (117.61 ± 41.97 mg/dL; r = 0.504; p < 0.0001). In contrast, hemoglobin (11.22 ± 2.10 g/dL; r = −0.242; p = 0.042), high-density lipoprotein cholesterol (HDL-C) (40 (28-57) mg/dL; ρ = -0.458; p = 0.0001), and serum albumin (3.4 (2.85-3.8) g/dL; ρ = −0.256; p = 0.031) showed significant negative correlations with the TyG index. No significant correlations were observed between the TyG index and the remaining biochemical parameters (Table 2).

**Table 2:** Biochemical parameters and their correlation with the TyG index HbA1c: glycated hemoglobin, SGOT: serum glutamic-oxaloacetic transaminase, AST: aspartate aminotransferase, SGPT: serum glutamic-pyruvic transaminase, ALT: alanine aminotransferase, eGFR: estimated glomerular filtration rate, HDL-C: high-density lipoprotein cholesterol, LDL-C: low-density lipoprotein cholesterol, NT-proBNP: N-terminal pro-B-type natriuretic peptide, SD: standard deviation, Rho: Spearman rank correlation coefficient, r: Pearson correlation coefficient, TG: triglycerides, TC: total cholesterol

Biochemical parameters	Mean ± SD (range)/median (25th-75th percentile)	rho/r	p-value
Fasting TG	170(127-237)	0.72	<0.0001
Fasting glucose	118(104.5-171.5)	0.881	<0.0001
HbA1c (%)	5.9(5.4-6.1)	0.348	0.003
Hemoglobin (g/dL)	11.22 ± 2.1 (4.1-16.5)	-0.242	0.042
Total leukocyte count (cells/µL)	9408.45 ± 3130.21 (3800-17600)	-0.079	0.512
Platelet count (×10³ cells/µL)	182(129-276.5)	0.092	0.446
Total bilirubin (mg/dL)	0.7(0.5-0.9)	0.024	0.84
Direct bilirubin (mg/dL)	0.1(0.1-0.2)	-0.135	0.26
SGOT/AST (U/L)	39(25.5-55.5)	-0.135	0.262
SGPT/ALT (U/L)	32(19.5-53.5)	-0.108	0.369
Serum albumin (g/dL)	3.4(2.85-3.8)	-0.256	0.031
Serum urea (mg/dL)	40(26.3-66)	-0.186	0.12
Serum creatinine (mg/dL)	0.93(0.695-1.4)	-0.13	0.279
eGFR (mL/min/1.73 m²)	76(49-99)	0.003	0.983
Serum sodium (mEq/L)	136.75 ± 6.1 (124-151)	-0.148	0.217
Serum potassium (mEq/L)	4.22 ± 0.67 (2.7-6)	0.013	0.912
TC (mg/dL)	167(135-221.5)	0.557	<0.0001
HDL-C (mg/dL)	40(28-57)	-0.458	0.0001
LDL-C (mg/dL)	117.61 ± 41.97 (30-209)	0.504	<0.0001
NT-proBNP (pg/mL)	432(212-960)	-0.031	0.796

Correlation of the TyG index with PLAN prognostic score

**Figure 1:**
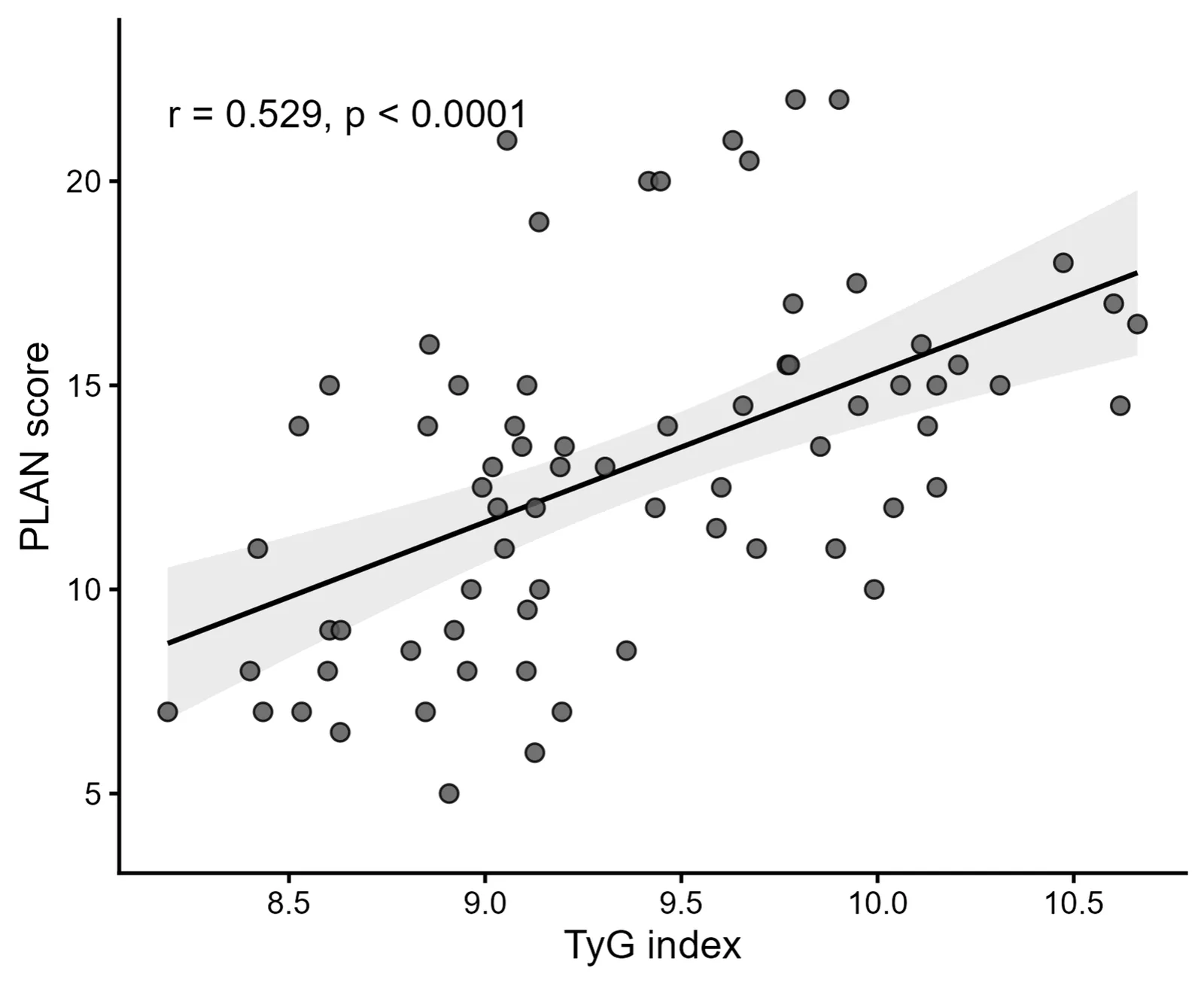
Correlation of the PLAN score with the TyG index r = 0.529, p < 0.0001 PLAN: Preadmission Comorbidities, Level of Consciousness, Age, and Neurological Deficit Score; TyG: triglyceride-glucose

A significant positive correlation was observed between the TyG index and the PLAN prognostic score (r = 0.529, p < 0.0001), as shown in Figure 1. After adjustment for age, sex, hypertension, diabetes mellitus, smoking, and heart failure, the correlation remained significant and of similar magnitude (partial r = 0.540, 95% CI: 0.342-0.693; p < 0.0001), indicating that the association was independent of these baseline characteristics.

**Figure 2:**
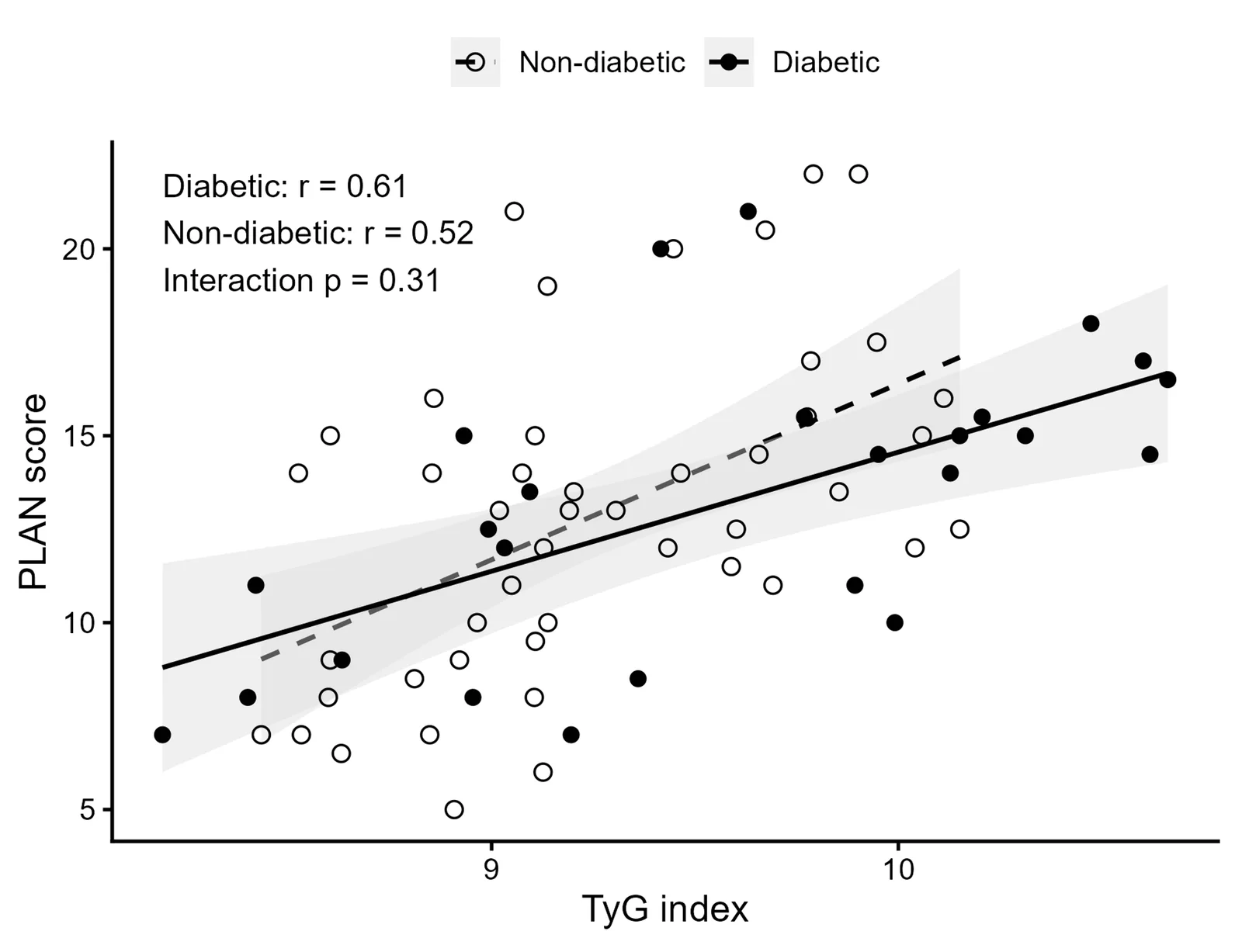
Correlation of the PLAN score with the TyG index by diabetes status Filled circles/solid line: diabetic patients, open circles/dashed line: non-diabetic patients, bands: 95% CI PLAN: Preadmission Comorbidities, Level of Consciousness, Age, and Neurological Deficit, TyG: triglyceride-glucose, CI: confidence interval

Furthermore, within both the diabetic and non-diabetic subgroups, the PLAN score showed a significant positive correlation with the TyG index. The correlation was moderately strong among diabetic patients (r = 0.608, 95% CI: 0.280-0.809; p = 0.001) and moderate among non-diabetic patients (r = 0.520, 95% CI: 0.271-0.704; p = 0.0002). A significant correlation was also observed in the overall cohort (r = 0.529, 95% CI: 0.338-0.679; p < 0.0001). However, the TyG-diabetes mellitus interaction was not significant (p = 0.306), indicating that the strength of the association between the TyG index and the PLAN score was comparable between diabetic and non-diabetic patients (Figure 2).

## Discussion

The present study demonstrated a significant positive correlation between the TyG index and the PLAN score, a validated prognostic scoring system for patients with AIS. This finding suggests that a higher TyG index may be associated with a greater likelihood of adverse outcomes. Similar observations have been reported by Zhou et al. and Lee et al., who found that elevated TyG index values were associated with neurological deterioration, higher National Institutes of Health Stroke Scale scores, poorer functional outcomes, and increased disability [10,11]. Furthermore, previous studies have reported a positive correlation between the TyG index and the Global Registry of Acute Coronary Events risk score, highlighting the prognostic significance of the TyG index in cardiovascular and cerebrovascular diseases [12,13].

In the present study, the TyG index showed significant positive correlations with lipid profile parameters (except for HDL-C, which showed an inverse relationship) and HbA1c. In contrast, a significant negative correlation was observed with hemoglobin levels. Similarly, Wan et al. reported significant trends in lipid parameters across increasing quartiles of the TyG index [14]. Higher TyG quartiles were associated with progressive increases in LDL-C, TG, and TC, whereas HDL-C levels declined significantly (p < 0.05). These findings are consistent with those reported by Deng et al. [15], who also observed strong correlations between the TyG index and lipid profile parameters.

Likewise, another study demonstrated significant positive associations between the TyG index and lipid parameters, except for HDL-C, which showed an inverse relationship [16]. Their study reported significant increases in TC and TG levels with increasing TyG index values, whereas HDL-C levels decreased significantly; however, the study was conducted in patients with cardiovascular disease. Zhou et al. also observed significant differences in lipid profiles across TyG quartiles, with a progressive increase in median triglyceride levels from the lowest to the highest quartile, indicating that elevated TyG index values are associated with an unfavorable lipid profile [10]. Similar findings were reported by Wang et al. [13], who demonstrated direct correlations between the TyG index and lipid profile parameters.

Additionally, Shi et al. highlighted the clinical significance of elevated TyG index values by demonstrating a 22.8% increase in the risk of ischemic stroke with increasing TyG index values [16]. Collectively, these findings support the observation that higher TyG index values are associated with adverse lipid profiles and an increased risk of stroke.

The association between the TyG index and dyslipidemia may be explained by insulin resistance, which promotes abnormal lipid metabolism and accelerates atherosclerotic changes in the cerebral vasculature. Atherosclerosis of the arteries supplying the brain is a major contributor to ischemic stroke. Therefore, the TyG index may serve as a simple and useful marker for identifying patients at increased risk of stroke [13,16].

Importantly, as a surrogate marker of insulin resistance, the TyG index showed a strong positive correlation with fasting blood glucose among AIS patients in the present study. The association between the TyG index and the PLAN score was observed in both the diabetic and non-diabetic groups, with no significant difference between groups. This finding emphasizes its clinical relevance not only in diabetic patients but also in the broader stroke population. Similar associations between the TyG index and impaired glucose metabolism have been reported by Wan et al. [14], Deng et al. [15], Selvi et al. [18], and Hong et al. [20].

Likewise, Zhou et al. observed progressively increasing fasting plasma glucose levels across TyG quartiles, suggesting a strong positive association between the TyG index and poor glycemic control [10]. Wang et al. also reported a significant positive correlation between the TyG index and fasting plasma glucose levels, indicating that elevated TyG index values are closely associated with hyperglycemia [13].

Overall, the findings of the present study, together with existing evidence, suggest that an elevated TyG index is strongly associated with impaired glycemic control and increased insulin resistance. Consequently, the TyG index may be a valuable, readily obtainable biomarker for assessing metabolic dysfunction and risk stratification in patients with AIS.

Limitations of the study

This study has certain limitations. First, it was conducted at a single tertiary care center with a relatively small sample size, which may limit the generalizability of the findings and statistical power. Second, the cross-sectional design precludes assessment of temporal relationships and does not allow causal inferences between the TyG index and the PLAN score. Third, the analysis of biochemical parameters was based on unadjusted bivariate correlations; therefore, potential confounding by age, sex, smoking status, and associated comorbidities cannot be excluded; however, the independent association of the TyG index with the PLAN score was established after adjusting for clinical confounders. Fourth, multiple correlation analyses were performed without adjustment for multiple comparisons, thereby increasing the possibility of type I error. Fifth, the study cannot establish an independent prognostic association between the TyG index and clinical outcomes, as longitudinal outcomes and multivariable prognostic analyses were not performed. Sixth, only baseline TyG measurements were assessed, limiting evaluation of temporal changes in metabolic status, and the relationship between the TyG index and established measures of insulin resistance, such as Homeostatic Model Assessment of Insulin Resistance (HOMA-IR), was not evaluated. Lastly, in the present study, medication history, including statin use, was not systematically recorded as a study variable; therefore, a subgroup analysis based on statin therapy could not be performed, although statins may affect the lipid profile. Similarly, specific cardiac risk markers and predefined cardiovascular risk criteria were not prospectively incorporated into the study protocol.

Strengths of the study

The study has several strengths. It comprehensively evaluated the association between the TyG index and the PLAN score, a validated prognostic tool for predicting adverse outcomes after AIS. Additionally, a broad range of biochemical parameters was analyzed, enabling a detailed assessment of the metabolic profile of AIS patients. The study also compared subgroups of diabetic and non-diabetic patients with respect to the TyG index, PLAN score, and baseline demographic characteristics.

Future recommendations

Future studies should include larger, multicenter, prospective cohorts with longitudinal assessment of the TyG index and clinically relevant stroke outcomes. Multivariable regression analyses should be performed to determine whether the TyG index is independently associated with prognostic outcomes after adjustment for potential confounders. Appropriate correction for multiple comparisons should also be considered when evaluating multiple biochemical associations. Serial TyG measurements and assessment of HOMA-IR or other established measures of insulin resistance may further clarify the metabolic and prognostic significance of the TyG index in patients with AIS. Lastly, future prospective studies should systematically record statin and other lipid-lowering therapies, antihyperglycemic medications, antihypertensive treatments, and other relevant medications, along with predefined cardiovascular risk markers. Such studies should incorporate these variables into multivariable analyses to determine whether the observed association between the TyG index and the PLAN score remains independent of treatment-related and cardiovascular risk factors.

## Conclusions

The TyG index showed a positive correlation with the PLAN prognostic score and several biochemical parameters among patients with AIS. Higher TyG index values were associated with an adverse lipid and glycemic profile, reflecting underlying metabolic abnormalities. These findings suggest that the TyG index may serve as a simple and readily available marker of metabolic abnormalities in patients with AIS. However, given the cross-sectional design, the present study cannot establish an independent prognostic or causal relationship between the TyG index and stroke outcomes. Further prospective, multicenter studies incorporating adjustment for potential confounders and longitudinal clinical outcomes are required to determine whether the TyG index provides independent prognostic information in AIS.
